# Epidemiological Characteristics of Respiratory Syncytial Virus in Pediatric Acute Lower Respiratory Tract Infections in Baoding, Hebei Province of China, from 2017 to 2024

**DOI:** 10.3390/children13060829

**Published:** 2026-06-18

**Authors:** Ju Yin, Su He, Xiao Zhang, Xiaomeng Liu, Baoping Xu, Yiqin Song

**Affiliations:** 1 Respiratory Department, Beijing Children’s Hospital, Capital Medical University, National Center for Children’s Health, Beijing 100045, China; yinju@bch.com.cn; 2 China National Clinical Research Center of Respiratory Diseases, Beijing 100045, China; 3 Respiratory Department, Baoding Hospital of Beijing Children’s Hospital, Capital Medical University, Baoding 071000, China; 15832289659@163.com (S.H.); liu17732203972@163.com (X.L.); 4 Clinical Laboratory, Baoding Hospital of Beijing Children’s Hospital, Capital Medical University, Baoding 071000, China; 13832296578@126.com; 5 Baoding Key Laboratory of Clinical Research on Children’s Respiratory and Digestive Diseases, Baoding 071000, China

**Keywords:** acute lower respiratory tract infection, respiratory syncytial virus, children, infants, epidemiology, coronavirus disease 2019 (COVID-19) pandemic, seasonality

## Abstract

**Highlights:**

**What are the main findings?**

- RSV remains the predominant pathogen for hospitalized ALRTI in Baoding, particularly among infants. During and after the COVID-19 pandemic, while the exact number of RSV-positive detections significantly decreased, the proportion of affected children aged ≥5 years was significantly higher than before the pandemic.
- Due to the impact of the COVID-19 pandemic and its subsequent effects, the seasonal patterns of RSV epidemics have undergone significant changes in the Baoding region, China. RSV seasonality shifted from a typical single winter–spring peak in the pre-COVID-19 era to a markedly reduced peak during the pandemic, in contrast to a delayed summer–autumn “off-season” pattern observed in the post-pandemic period.

**What are the implications of the main findings?**

- An in-depth analysis of the epidemiological characteristics of RSV at the provincial geographic division level in China is of significance for the formulation of regional prevention strategies against RSV infection.
- The importance of establishing RSV surveillance at both the national and subnational levels in China should be emphasized.

**Abstract:**

**Objectives**: To investigate the clinical and epidemiological characteristics of respiratory syncytial virus (RSV)-associated acute lower respiratory tract infection (ALRTI) among hospitalized children in the Baoding region of Hebei Province, China. **Methods**: The study subjects were inpatients diagnosed with ALRTI at a pediatric hospital in Baoding between 1 June 2017 and 31 December 2024. A retrospective analysis was conducted on the detection of RSV and other common respiratory viruses in respiratory specimens to evaluate the clinical epidemiological characteristics of RSV. Viral detection was performed using immunofluorescence (IF) or polymerase chain reaction (PCR) assays. **Results**: The overall RSV detection rate was 16.8% (4945/29,399), with 6.7% co-infections. The RSV detection rate was 29.8% (2604/8729) in infants younger than 1 year and 18% (942/5232) in the 1-year-old group, then showing a decreasing trend with increasing age, with the lowest rate of 3.8% (283/7366) observed in the ≥5 years age group. The RSV detection rate decreased from 19.9% before the COVID-19 pandemic (2017–2019) to around 13% during and after the pandemic (χ^2^ = 233.732, *p* < 0.001), accompanied by a drop in the proportion of children under 2 years from 78.5–79.6% before the COVID-19 pandemic to 42.1–51.1% after the pandemic period (χ^2^ = 387.565, *p* < 0.001). RSV seasonality shifted from a typical single winter–spring peak in the pre-COVID-19 era to a markedly reduced peak during the pandemic, in contrast to a delayed summer–autumn “off-season” pattern observed in the post-pandemic period. **Conclusions**: RSV remains the predominant pathogen for hospitalized ALRTI in Baoding, particularly among infants. Detection rates of RSV declined with increasing age. The COVID-19 pandemic has profoundly impacted RSV prevalence patterns and seasonality, underscoring the critical need for long-term surveillance.

## 1. Introduction

Respiratory syncytial virus (RSV) is a major pathogen of acute lower respiratory tract infection (ALRTI) in children under 5 years of age, accounting for nearly 30% of all respiratory pathogens [1]. Globally, an estimated over 33 million episodes of RSV-associated ALRTI occurred in children under 5 years in 2019, of which approximately 3.6 million required hospitalization, and about 95% of these episodes occurred in low- and middle-income countries [2]. In China, ALRTI is the infectious disease with the highest mortality rate among children and adolescents [2,3], approximately 40.3 cases of RSV-associated LRI per 1000 children occur annually [1], and an estimated 620,000 to 950,000 hospitalizations for RSV-associated respiratory infections occur in children under 5 years of age [4]. In recent years, nirsevimab, a monoclonal antibody to the RSV fusion protein, has been approved for use in newborns and infants, significantly reducing the disease burden of RSV-associated ALRTI [5]; however, its protective effect lasts only 5–6 months, necessitating its use in eligible children who are about to enter, or are already in, the RSV season. Therefore, it is crucial to clarify region-specific seasonal patterns of RSV epidemiology.

Given China’s vast territory, the seasonal epidemiology of RSV exhibits a distinct latitudinal gradient, with varying epidemic patterns across different regions [6]. However, the coronavirus disease 2019 (COVID-19) pandemic has further disrupted the epidemic patterns of RSV [7,8,9]. Therefore, an in-depth analysis of RSV epidemiological characteristics in various regions of China in the post-pandemic era is of great significance for formulating region-specific RSV prevention strategies [10].

This study aims to analyze the epidemiological characteristics and seasonal distribution patterns of RSV-associated ALRTI in the Baoding region of Hebei Province over a continuous period of 7.5 years, from June 2017 to December 2024, in order to provide fundamental data and a reference basis for the development of evidence-based RSV prevention and control strategies in this region.

## 2. Materials and Methods

### 2.1. Study Subjects

Children (0~17 years of age) hospitalized due to ALRTI at Baoding Hospital of Beijing Children’s Hospital from 1 June 2017 to 31 December 2024 were enrolled. ALRTI included clinically diagnosed tracheitis, bronchitis, bronchiolitis, or pneumonia, based on the diagnostic criteria of Zhu Futang Practical Pediatrics, 9th Edition [11]. Demographic and clinical data were obtained from the hospital’s electronic medical record system.

### 2.2. Specimens and Viral Detection

Viral antigen detection was performed using antigen detection kits provided by Diagnostic Hybrids, Inc. Athens, OH, USA. Direct immunofluorescence (IF) assay was used to detect respiratory viral antigens, including RSV, parainfluenza virus (PIV) types 1, 2, and 3, influenza virus A (IFV-A), influenza virus B (IFV-B), and adenovirus (ADV). Results were observed under an Olympus BX51 fluorescence microscope (Evident Corporation Inc, Tokyo, Japan). A specimen was considered positive if bright green fluorescence was observed in at least two intact cells under high-power magnification; otherwise, it was considered negative. From 2017 to 2020, all specimens were tested using the IF method. Since 2021, nucleic acid testing has been performed by using a kit provided by Ningbo Health Gene Technologies Co., Ltd., Ningbo, Zhejiang Province, China. PCR capillary electrophoresis fragment analysis was used to detect 9 respiratory viruses [including RSV, PIV, IFV-A, IFV-B, ADV, rhinovirus (RV), bocavirus (BoV), metapneumovirus (MPV), and coronavirus (CoV)-OC43], which expanded the range of detectable viruses, and the use of IF gradually decreased. Overall, 20,268 specimens were detected by IF and 9133 by PCR.

### 2.3. Ethics Approval

This study was approved by the Ethics Committee of Baoding Children’s Hospital [Approval No.: 2025 (Fast Review) [Science] No. (10)]. Anonymized data were used in this study, which did not involve patient privacy or commercial interests.

### 2.4. Statistical Analysis

Statistical analyses were performed using SPSS version 26.0. Categorical variables were expressed as frequencies and percentages. The Pearson chi-square test was used for intergroup comparisons. A *p*-value < 0.05 was considered statistically significant.

## 3. Results

### 3.1. Annual Distribution and Age Composition of Hospitalized Children with ALRTI

**Figure 1:**
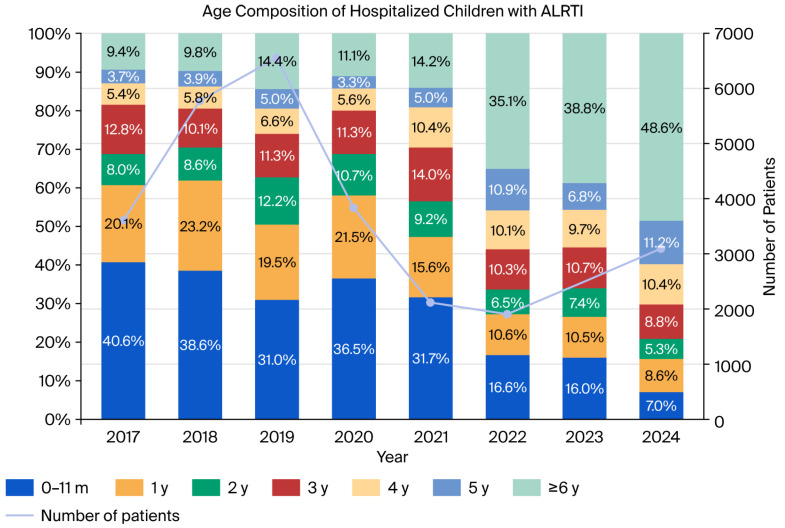
Annual distribution and age composition of hospitalized children with ALRTI. Note: Data for 2017 were collected from June to December (7 months), while data for the other years were collected from the full 12-month period.

From 1 June 2017 to 31 December 2024, a total of 29,399 children hospitalized with ALRTI were enrolled in this study, including 17,333 males and 12,066 females, with a male-to-female ratio of 1.44:1. The age ranged from 20 h to 16 years, with a median age of 2 years. The annual age composition is detailed in Figure 1.

According to the year of hospitalization, during the pre-COVID-19 pandemic period (2017–2019, 2.5 years), a total of 15,943 children were hospitalized, with the highest number of 6556 cases occurring in 2019. During the pandemic period (2020–2022, 3 years), 7861 cases were enrolled; the lowest number of 1908 cases occurred in 2022, which was below the pre-pandemic level. During the post-pandemic period (2023–2024, 2 years), 5595 cases were enrolled, showing a rebound in the average annual number of cases compared to the pandemic period, though it had not yet reached the pre-pandemic level.

During the study period, the age composition of hospitalized children with ALRTI changed significantly. From 2017 to 2019, infants and young children aged 0–2 years predominated, accounting for 68.7–70.4%, while children aged ≥5 years accounted for 13.1–19.4%. From 2020 to 2022, the proportion of children aged 0–2 years decreased from 68.7% in 2020 to 33.7% in 2022, whereas the proportion of children aged ≥5 years increased from 14.4% to 46.0%. From 2023 to 2024, the proportion of children aged 0–2 years further declined, ranging from 33.9% in 2023 to 20.9% in 2024, while children aged ≥5 years accounted for 45.6–59.9% over the same period. Overall, the age structure shifted from being dominated by infants and toddlers before the pandemic to being dominated by school-aged children after the pandemic.

### 3.2. RSV Positive Detection and Age Distribution Among Hospitalized Children with ALRTI

Among 29,399 children with ALRTI, RSV was detected as positive in 4945 cases, resulting in an overall positive detection rate of 16.8%. The male-to-female ratio was 1.48:1 (2949 to 1996). A total of 330 cases tested positive for two or more viruses, yielding a co-infection rate of 6.7% (330/4945).

The RSV positive detection rate showed a declining trend with increasing age. The positive detection rate in the <5 years age group was 21.1% (4662/22,033). Specifically, it was 44.5% (929/2088) in infants aged 0–2 months, 31.2% (742/2381) in those aged 3–5 months, and 21.9% (933/4260) in those aged 6–11 months. The rate was 18.0% (942/5232) in the 1 year age group. The lowest positive detection rate was observed in the ≥5 years age group, at 3.8% (283/7366) (Table 1).

**Figure 2:**
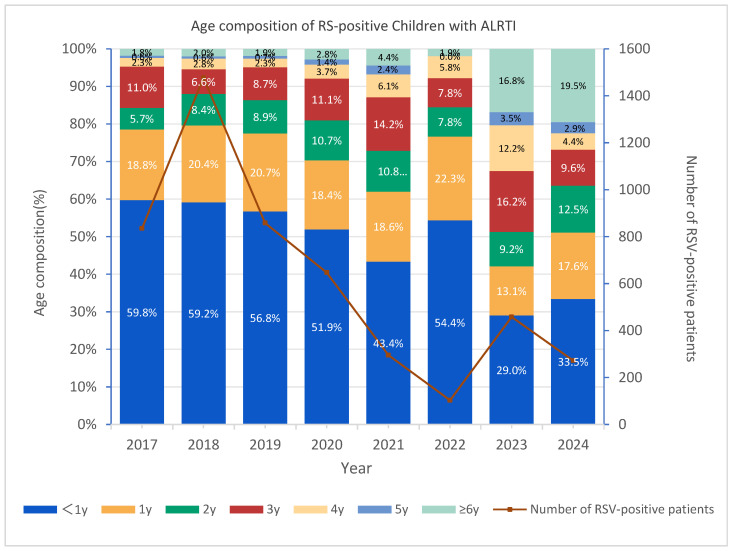
Annual distribution and age composition of RSV-positive children with ALRTI.

Throughout the study period, RSV-positive ALRTI cases were predominantly younger children; however, the age distribution varied across different years. From 2017 to 2019, the 0–2 years age group accounted for 84.3–88.0%, the 3–4 years group for 9.4–13.3%, and the ≥5 years group for 2.4–2.6%. From 2020 to 2022, the 0–2 years group accounted for 72.9–84.5%, the 3–4 years group for 13.6–20.3%, and the ≥5 years group for 1.9–6.8%. In 2023–2024, the 0–2 years group accounted for 51.3–63.6%, the 3–4 years group for 14.0–28.4%, and the ≥5 years group for 20.3–22.4% (χ^2^ = 387.565, *p* < 0.001). Thus, during and after the COVID-19 pandemic, while the absolute number of RSV-positive detections significantly decreased, the proportion of affected children in the 3–4 years age group increased. This proportion further rose in the post-COVID period, and the proportion of children aged ≥5 years was also significantly higher than before the pandemic (Figure 2).

### 3.3. Seasonal Characteristics of RSV Positive Detection Rates in Hospitalized Children with ALRTI

**Figure 3:**
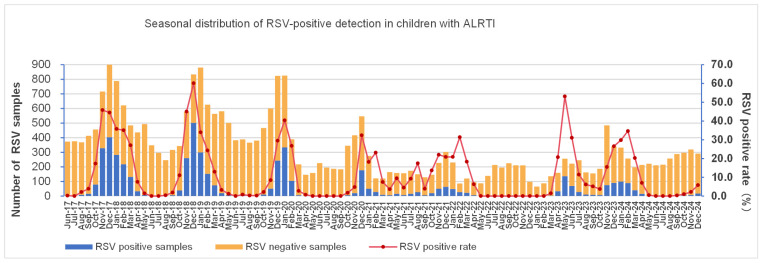
Seasonal distribution of RSV positive detection in children with ALRTI.

The seasonal fluctuation and phased epidemic characteristics of RSV infection during the study period are shown in Figure 3. From 2017 to 2019, RSV infection exhibited a typical single-peak winter epidemic pattern, with the high-incidence period occurring from October to March of the following year and the peak usually appearing in December or January. During the 2018–2019 epidemic season, the highest numbers of RSV-positive cases in the entire study period were detected in December, with a positive detection rate exceeding 60% (501/833), indicating that RSV was the predominant pathogen of ALRTI at that time. Viral activity was low in summer (June–August), with positive detection rates generally below 5%.

From 2020 to 2022, the traditional seasonal pattern persisted in winter and spring, but the magnitude of the peak decreased dramatically, and sporadic cases were observed in summer and autumn. Although RSV-positive case counts and positive detection rates in December 2020 and December 2021 remained higher than in other months of the same year, the number of positive cases was only approximately 50% of the pre-pandemic level, and the positive detection rate dropped to 32.4%. From May 2022 to February 2023, no RSV cases were detected, meaning no epidemic peak occurred in December 2022. Instead, an “off-season” outbreak emerged from March to July 2023 (spring–summer), with the monthly positive detection rate rising to over 50%, approaching the pre-pandemic winter peak level; however, the number of positive specimens was far lower than number before the pandemic.

From November 2023 (winter) to April 2024 (spring), an RSV epidemic peak was observed, though it had not yet returned to the pre-pandemic level. Since September 2024, sporadic RSV detections have appeared, and no positive detection peak had been observed as of December 2024, suggesting that the 2024 winter epidemic had been delayed, compared to pre-pandemic seasonal patterns.

In summary, RSV seasonality shifted from a typical single winter–spring peak in the pre-COVID-19 era to a markedly reduced peak during the pandemic, in contrast to a delayed summer–autumn “off-season” pattern observed in the post-pandemic period.

### 3.4. Pathogen Detection in RSV Co-Infections Among Hospitalized Children with ALRTI

A total of 330 RSV co-infection cases were identified among the hospitalized pediatric patients with ALRTI in this study. Regarding age distribution, children under 5 years of age accounted for 83.0% (274/330), and children under 2 years accounted for more than half (191/330, 57.9%).

An analysis based on detection methods and time periods showed that from 2017 to 2020, immunofluorescence assays were used, identifying 113 co-infection cases with a co-infection rate of 3.0% (113/3817). The pathogen spectrum included influenza virus (IFV), parainfluenza virus (PIV), and adenovirus (ADV). From 2021 to 2024, PCR-based methods were predominantly used, identifying 217 co-infection cases with a rate of 19.23% (217/1128)—approximately five times higher than with the immunofluorescence assay. In addition to IFV, ADV, and PIV, the detected pathogens also included rhinovirus (RV), bocavirus (BoV), metapneumovirus (MPV), and CoV-OC43.

In terms of the number of co-infecting viral species (see Table 2), 305 cases involved two viruses, 24 cases involved three viruses, and 1 case involved four viruses. The predominant co-infection types were, in order, RSV + IFV in 80 cases (24.2%), RSV + ADV in 78 cases (23.6%), RSV + RV in 60 cases (18.2%), and RSV + PIV in 44 cases (13.3%). Among the 25 cases with co-infections of three or more viral species, 19 cases (76.0%) occurred between 2021 and 2024.

## 4. Discussion

This retrospective study analyzed the epidemiological characteristics of RSV-associated acute lower respiratory tract infection (RSV-ALRTI) among children in the Baoding region from June 2017 to December 2024, spanning 7.5 years and covering three distinct periods: pre-COVID-19, during the COVID-19 pandemic, and post-COVID-19.

RSV is the most frequently detected virus in ALRTI among children under 5 years of age, with the highest detection rate observed in the 0–6 months age group [12,13]. In the present study, the overall RSV detection rate among children with ALRTI was 16.8%. The highest RSV positive detection rate was 44.5% in infants aged 0–2 months, followed by 31.2% in those aged 3–5 months. The positive detection rate gradually decreased with increasing age, reaching only 3.8% in children aged ≥5 years. These findings are consistent with the results of a national multicenter study conducted by Zhu Yun et al. [14]. Thus, young infants are at high risk for severe RSV infection due to their immature immune system and impaired viral clearance capacity, highlighting the need for close monitoring and prevention.

During the pre-COVID-19 pandemic period (2017–2019), the majority of hospitalized children with ALRTI were infants and young children, with children aged 0–2 years accounting for 62.7–70.4% and children aged ≥5 years accounting for 13.1–19.4%. Regarding RSV-positive children hospitalized with ALRTI, over 80% were under 2 years of age, children aged 3–4 years accounted for 9.4–13.3%, and children aged ≥5 years accounted for only 2.4–2.6%. The epidemic season was concentrated from October–November to March–April of the following year, presenting as a single winter–spring peak, which is consistent with the seasonal pattern of RSV circulation in the Northern Hemisphere [6].

During the COVID-19 pandemic (2020–2022), the annual number of pediatric patients hospitalized with ALRTI decreased by over 50% compared to the pre-pandemic period. In terms of age distribution, the proportion of infants and toddlers aged 0–2 years declined (from 68.7% to 33.7%), while the proportion of children aged ≥5 years increased (from 14.4% to 46.0%). The RSV positive detection rate among hospitalized ALRTI patients was 13.3%, significantly lower than the pre-pandemic rate of 19.9%. Within the RSV-positive ALRTI cases, the 0–2 years age group accounted for 72.9–84.5%, the 3–4 years group for 13.6–20.3%, and the ≥5 years group for 1.9–6.8%. This indicates a shift in the age distribution of RSV-positive ALRTI patients, with an increase in preschool-aged children and a decrease in infants and toddlers. Regarding the seasonal epidemiological characteristics of RSV, although the pattern remained predominantly in winter and spring in 2020, a minor peak also emerged during the spring and summer seasons. From April to the end of December 2022, the RSV positive detection rate was nearly zero. This phenomenon can be attributed to the non-pharmaceutical interventions (NPIs)—such as mask wearing, social distancing, and limiting gatherings—implemented globally following the onset of the COVID-19 pandemic in 2020 to curb the transmission of severe acute respiratory syndrome coronavirus 2 (SARS-CoV-2). These measures not only blocked the spread of SARS-CoV-2 but also suppressed the transmission of other respiratory viruses [15,16].

During 2023–2024 (post-pandemic period), the average annual number of hospitalized children with ALRTI increased compared with that during the pandemic period but remained significantly lower than the pre-pandemic level. In terms of the age composition of ALRTI cases, the proportion of children aged ≥5 years increased markedly to approximately 50%, while the proportion of infants and young children decreased further. Children with RSV-positive ALRTI shifted from an infant-dominated pattern before the pandemic to a school-age child-dominated pattern after the normalization of pandemic prevention and control, which is consistent with reports from Suzhou and other regions in China as well as overseas studies [9,17,18,19]. Research from the United States has shown that after the normalization of prevention and control, the growth rate of ALRTI among children aged 2–4 years was higher than that in infants aged 0–5 months [17].

In this study, the positive detection rate of RSV among ALRTI children during the post-pandemic period was 13.0%, which was similar to that during the pandemic period. There was no sharp surge in the total number of RSV infection cases associated with the so-called “immunity debt” [20], as reported domestically and internationally [8,17,18,21]. This may be attributed to geographical location, climate, latitude, and other factors as well as the prevalence of new RSV subtypes [9]. In terms of the seasonal epidemiological characteristics of RSV, a bimodal epidemic pattern was observed in 2023, with the first peak occurring from April to July and the second from November 2023 to April 2024. No RSV epidemic was observed thereafter until December 2024, presenting a lagged epidemic season [22]. The above findings suggest that in Baoding region, the epidemic pattern of RSV infection has not returned to the pre-pandemic unimodal winter–spring peak within two years after the normalization of pandemic prevention and control, and no increase in infections has been seen in young infants. This change in age structure may be related to the large-scale epidemic of *Mycoplasma pneumoniae* pneumonia in northern China after the normalization of prevention and control of COVID-19 [23], which led to a higher proportion of hospitalized school-age children with ALRTI. In addition, it is noteworthy that the number of newborns in Baoding has shown a downward trend in recent years [24]: approximately 100,000 births in 2018, dropping to 63,800 in 2021 and 49,400 in 2023. This may partly account for the relatively lower number of hospitalized infants and young children as well as RSV infections after 2022.

Co-infection with multiple pathogens is common in children with ALRTI, although the causal relationship between pathogens and clinical diseases requires individualized analysis [21,25]. In this study, a total of 330 RSV-positive children were co-infected with one or more additional respiratory viruses. The most prevalent co-infecting viruses were influenza virus (24.2%, 80/330), adenovirus (23.6%, 78/330), and rhinovirus (18.2%, 60/330), which is consistent with previous studies [26]. The spectrum of RSV mixed infections changed alongside the update to detection technologies during the study period. In the early phase (2017–2020), immunofluorescence assay was applied to detect influenza virus, adenovirus and parainfluenza virus. In the later phase (2021–2024), PCR was used for viral nucleic acid testing, adding four more pathogens: rhinovirus, bocavirus, metapneumovirus, and coronavirus. Due to the differences in detection methods and the range of screened pathogens, the mixed infection rate was 3.0% (113/3817) from 2017 to 2020 and rose significantly to 19.2% between 2021 and 2024, with an obvious increase in both the quantity and variety of detected viruses. These findings are in line with the results reported by Ferreira et al. [27]. The PCR assay yielded a substantially higher viral positive detection rate than the immunofluorescence assay (88.7% vs. 27.4%, *p* < 0.0001). Therefore, PCR testing presents the advantages of high efficiency and broad pathogen coverage. It can facilitate accurate clinical diagnosis and provide comprehensive epidemiological data for the formulation of therapeutic strategies and public health policies.

The limitations of this study are as follows: First, the study subjects were exclusively hospitalized pediatric patients with ALRTI who tested positive for RSV, which may not fully represent the overall epidemiological characteristics of RSV infection. Second, limitations in detection methods and the range of pathogens tested restricted a comprehensive analysis of the pathogens [28]. Due to the long study duration, antigen detection methods were used during the initial four years; these methods have lower sensitivity and specificity compared to nucleic acid testing, which may have led to an underestimation of viral detection rates and co-infection rates in ALRTI in the early stage of the study. Third, this study did not perform RSV subtyping or genomic analysis, making it impossible to more precisely characterize the overall differences in RSV epidemiology [9,23]. Fourth, this study included patients from only a single medical institution, which may introduce certain selection bias. However, as the only tertiary specialized children’s hospital in the Baoding region, its service network covers 22 surrounding counties and cities. With 1000 beds and a large annual admission volume for pediatric lower respiratory tract infections, it is well-positioned to represent the characteristics of the pediatric population in this region.

## 5. Conclusions

In conclusion, through a continuous 7.5-year analysis, this study described the epidemiological characteristics of RSV-ALRTI in the Baoding region across three periods: pre-, during, and post-COVID-19. RSV is a predominant pathogen of ALRTI among children (especially infants and toddlers) in Baoding. The RSV positive detection rate among ALRTI patients is age-dependent. The implementation of large-scale NPIs during the COVID-19 pandemic likely significantly altered its epidemiological patterns, with effects persisting up to two years post-pandemic. Future surveillance at both the national and subnational levels is needed to monitor the ongoing trends.

## Figures and Tables

**Table 1:** Characteristics of RSV-positive cases among hospitalized children with ALRTI.

	Number of ALRTI Cases	Number of RSV-Positive	Positive Rate	χ^2^	*p* Value
Sex				1.130	0.288
Male	17,333	2949	17.0%		
Female	12,066	1996	16.5%		
Age				2047.929	0.000
<1 y	8729	2604	29.8%		
0–2 m	2088	929	44%		
3–5 m	2381	742	31.2%		
6–11 m	4260	933	21.9%		
1 y	5232	942	18.0%		
2 y	2663	433	16.3%		
3 y	3254	486	14.9%		
4 y	2155	197	9.1%		
5 y	1646	60	3.6%		
≥6 y	5720	223	3.9%		
Year				233.732	<0.001
2017–2019	15,943	3170	19.9%		
2020–2022	7861	1045	13.3%		
2023–2024	5595	730	13.0%		
Total	29,399	4945	16.8%		

**Table 2:** Pathogen distribution of RSV-positive co-infections in hospitalized children with ALRTI.

Year	2017	2018	2019	2020	2021	2022	2023	2024	Total
RSV-positive sample	835	1477	858	647	295	103	458	272	4945
Co-infections	8	24	61	20	53	4	88	72	330
Co-infection rate	1.0%	1.6%	7.1%	3.1%	18.0%	3.9%	19.2%	26.5%	6.7%
+ IFV	4	13	16	6	2	1	20	18	80
+ ADV	1	3	33	9	0		18	14	78
+ RV	-	-	-	-	23	3	19	15	60
+ PIV	3	8	6	5	4		10	8	44
+ Boca	-	-	-	-	20		7	1	28
+ MPV	-	-	-	-	0		2	6	8
+ OC43					1		4	2	7
+ IFV + ADV			5					3	8
+ IFV + MPV								1	1
+ IFV + PIV							3		3
+ ADV + PIV							3	1	4
+ ADV + MPV								2	2
+ RV + ADV								1	1
+ RV + Boca					3		1		4
+ RV + MPV							1		1
+ IFV + ADV + PIV			1						1

## Data Availability

Data are presented within the article.

## Abbreviations

The following abbreviations are used in this manuscript:

ALRTI	acute lower respiratory tract infection
RSV	respiratory syncytial virus
COVID-19	coronavirus disease 2019
NPIs	non-pharmaceutical interventions
IF	immunofluorescence
PCR	polymerase chain reaction
IFV	influenza virus
ADV	adenovirus
RV	rhinovirus
BoV	bocavirus
PIV	parainfluenza virus
MPV	metapneumovirus
CoV	coronavirus
